# Metabolomic Associations with Serum Bone Turnover Markers

**DOI:** 10.3390/nu12103161

**Published:** 2020-10-16

**Authors:** Moriah P. Bellissimo, Joseph L. Roberts, Dean P. Jones, Ken H. Liu, Kaitlin R. Taibl, Karan Uppal, M. Neale Weitzmann, Roberto Pacifici, Hicham Drissi, Thomas R. Ziegler, Jessica A. Alvarez

**Affiliations:** 1 Division of Endocrinology, Metabolism and Lipids, Department of Medicine, Emory University School of Medicine, Atlanta, GA 30322, USA; Kaitlin.rene.taibl@emory.edu (K.R.T.); mweitzm@emory.edu (M.N.W.); roberto.pacifici@emory.edu (R.P.); tzieg01@emory.edu (T.R.Z.); jessica.alvarez@emory.edu (J.A.A.); 2 Emory Center for Clinical and Molecular Nutrition, Emory University, Atlanta, GA 30322, USA; dpjones@emory.edu; 3 Department of Orthopaedics, Emory University School of Medicine, Atlanta, GA 30322, USA; joseph.roberts@emory.edu (J.L.R.); hicham.drissi@emory.edu (H.D.); 4 Atlanta Department of Veterans Affairs Medical Center, Decatur, GA 30033, USA; 5 Division of Pulmonary, Allergy, Critical Care and Sleep Medicine, Department of Medicine, Emory University School of Medicine, Atlanta, GA 30322, USA; ken.liu@emory.edu (K.H.L.); karan.uppal@emory.edu (K.U.); 6 Emory Microbiome Research Center, Emory University, Atlanta, GA 30322, USA

**Keywords:** bone, metabolism, microbiome, nutrition, osteoclast, osteoblast

## Abstract

Bone is a dynamic tissue that is in a constant state of remodeling. Bone turnover markers (BTMs), procollagen type I N-terminal propeptide (P1NP) and C-terminal telopeptides of type I collagen (CTX), provide sensitive measures of bone formation and resorption, respectively. This study used ultra-high-resolution metabolomics (HRM) to determine plasma metabolic pathways and targeted metabolites related to the markers of bone resorption and formation in adults. This cross-sectional clinical study included 34 adults (19 females, mean 27.8 years), without reported illnesses, recruited from a US metropolitan area. Serum BTM levels were quantified by an ELISA. Plasma HRM utilized dual-column liquid chromatography and mass spectrometry to identify metabolites and metabolic pathways associated with BTMs. Metabolites significantly associated with P1NP (*p* < 0.05) were significantly enriched in pathways linked to the TCA cycle, pyruvate metabolism, and metabolism of B vitamins important for energy production (e.g., niacin, thiamin). Other nutrition-related metabolic pathways associated with P1NP were amino acid (proline, arginine, glutamate) and vitamin C metabolism, which are important for collagen formation. Metabolites associated with CTX levels (*p* < 0.05) were enriched within lipid and fatty acid beta-oxidation metabolic pathways, as well as fat-soluble micronutrient pathways including, vitamin D metabolism, vitamin E metabolism, and bile acid biosynthesis. P1NP and CTX were significantly related to microbiome-related metabolites (*p* < 0.05). Macronutrient-related pathways including lipid, carbohydrate, and amino acid metabolism, as well as several gut microbiome-derived metabolites were significantly related to BTMs. Future research should compare metabolism BTMs relationships reported here to aging and clinical populations to inform targeted therapeutic interventions.

## 1. Introduction

Bone is a dynamic tissue that is continually resorbed and formed through a process called remodeling [1,2]. Through the coordinated activities of bone-forming osteoblasts and bone-resorbing osteoclasts, adult bone mass is maintained at a relatively constant state until the rate of resorption outpaces that of formation [1,3]. This shift typically begins after attainment of peak bone mass around the age of 30 years and results in a gradual decline in bone mass from that point [4]. Preventing the progression of bone mass loss to osteoporosis is a public health priority because osteoporosis leads to a diminished quality of life coupled with disability and morbidity [5,6]. The metabolic activity of bone can be monitored by measuring the serum levels of bone turnover markers (BTMs) [7]. The International Osteoporosis Foundation and International Federation of Clinical and Laboratory Medicine have identified procollagen type I N-terminal propeptide (P1NP) and C-terminal telopeptides of type I collagen (CTX) as clinically relevant biomarkers of bone metabolism that capture sensitive changes in bone formation and bone resorption, respectively [8].

P1NP is the by-product of post-translational N-terminal cleavage of type 1 procollagen by osteoblasts, which is released into systemic circulation during periods of new bone formation [8]. Conversely, CTX is released into the systemic circulation during bone resorption as a result of the osteoclast-mediated hydrolysis of type I collagen stored in bone [9,10]. Both serum P1NP and CTX are responsive to interventions designed to prevent bone loss and can provide valuable insights into the effectiveness of anti-osteoporosis therapies before changes in bone mineral density (BMD) can be detected [8,11].

Shifts in bone metabolism and, subsequently, bone mass induce systemic metabolic changes. In addition to endogenously derived metabolites, the intestinal microbiota produce many bioactive compounds that can act as key modulators of bone metabolism when absorbed [12,13]. High-resolution metabolomics (HRM) offers a unique approach to acquire an unbiased snapshot of these endogenously and microbiota-derived systemic metabolites as well as overall metabolism related to a health outcome such as BTMs [14]. We previously reported that microbiome-related tryptophan and phenylacetic acid, as well as linoleic acid and its oxidized derivatives, were related to BMD in middle-aged adults [15]; however, the specific impact on sensitive bone turnover biomarkers is not clear. The objective of this study was to characterize the associations of P1NP and CTX concentrations with plasma metabolic pathways using unbiased HRM in a sample of healthy, young adults within the age range of peak bone mass accrual. A secondary aim was to conduct targeted analyses of microbiome-related and linoleic acid metabolites in relation to the BTMs.

## 2. Materials and Methods

### 2.1. Participants and Study Design

This cross-sectional study included 34 adults without known illness. Subjects were recruited to participate in the study through the use of flyers posted around the university area or word of mouth. Inclusion criteria were being 18 years of age or older, having no acute illness or hospitalization within the previous year, and able to walk without assistance. Exclusion criteria were having a current diagnosis of a chronic respiratory disease, chronic infection, pro-inflammatory or autoimmune disease, metabolic disease, or current active malignant neoplasm other than localized basal cancer of the skin, currently taking medications that may alter body composition, currently pregnant or breastfeeding, having a history of recreational or prescription drug or alcohol abuse, or weight instability in the previous six months (>10% change in body weight). The study was approved by the Emory University Institutional Review Board (IRB00085327; approval date 24 February 2020). All participants provided written informed consent prior to undergoing any study procedures.

### 2.2. Demographic and Clinical Information

Demographic information including sex and race, as well as current supplement use, was collected by self-reported questionnaires. All study subjects underwent a total body composition scan assessed by dual-energy X-ray absorptiometry (DXA, GE Lunar iDXA densitometer, GE Healthcare, Madison, WI, USA) to determine whole-body BMD, whole-body T-score, and percent body fat. A manual stadiometer was used to assess height after participants removed their shoes, and height was recorded to the nearest tenth of a centimeter. Weight was measured using a digital scale and recorded to the nearest tenth of a kilogram. Body mass index (BMI) was calculated as the weight in kilograms divided by the height in meters squared (kg/m^2^).

### 2.3. Assessment of Serum P1NP and CTX

Fasting blood samples were obtained from study participants in the morning following an overnight fast. Serum was isolated, aliquoted, and stored at −80 °C until used. The serum levels of total P1NP were quantified using a sandwich ELISA following the manufacturer’s instructions (MyBioSource, San Diego, CA, USA #MBS2504819; intra-assay CV of 13.3%). The serum levels of C-terminal telopeptides of Type I collagen (CTX-I) were quantified using the serum CrossLaps ELISA according to the manufacturer’s instructions (Immunodianostic Systems, Gaithersburg, MD, USA, IDS #AC-02F1; intra-assay CV 4.5%).

### 2.4. Plasma High-Resolution Metabolomics

HRM was performed at Emory University in the Clinical Biomarkers Laboratory as previously described [16]. Fasting plasma samples were collected in EDTA tubes, immediately placed on ice, and centrifuged to isolate plasma, and stored at −80 °C until analysis. Thawed fasting plasma samples (65 μL) were treated with 130 μL acetonitrile and an internal standards mixture of eight stable isotopes acquired from Cambridge Isotope Laboratories [17]. Samples were mixed and incubated for 30 min on ice, centrifuged at 16,000× *g* at 4 °C for 10 min to precipitate proteins, then transferred to autosampler vials and kept at 4 °C for the entirety of the analysis. Samples were analyzed in triplicate using a Thermo Q-Exactive HF high-resolution mass spectrometer (Thermo Fisher, Waltham, MA, USA) and Dionex Ultimate 3000 ultra-high-performance liquid chromatography (UHPLC, Dionex, Sunnyvale, CA, USA). Analyte separation was performed with hydrophilic interaction liquid chromatography (HILIC, Waters XBridge BEH Amide XP HILIC column, 2.1 × 50 mm^2^, 2.6 μm particle size) in positive electrospray ionization (ESI, HILIC/ESI+) mode, as well as reverse phase (C18, Higgins Targa C18 2.1 × 50 mm^2^, 3 μm particle size) chromatography in negative ESI mode (C18/ESI-). The details of separation parameters including mobile phase compositions and flow rates have been previously published [18]. The mass spectrometer was operated in full scan mode at 120,000 resolution and a scan range of 85 to 1275 mass-to-charge ratio (*m*/*z*). Pooled plasma reference samples were included at the beginning, middle, and end of each batch for quality control. A feature table with accurate *m*/*z*, retention time (RT, seconds) and metabolite intensities was produced from the raw data files using the validated data extraction programs apLCMS and xMSanalyzer [19,20]. Data extraction and pre-processing included peak detection and alignment, parameter optimization, and quality assessment and data correction. Batch correction was completed using ComBat [21]. The pre-processing of the metabolomic features was based on: (1) the filtering of features based on the coefficient of variation (CV); (2) filtering of samples based on the Pearson correlation between technical replicates; (3) averaging of technical replicates; (4) filtering of missing features (a feature was retained for downstream analysis only if present in ≥80% of samples); (5) log2 transformation and quantile normalization to improve biological interpretation and reduce the impact of variation on statistical analysis. A total of 13,353 metabolic features from HILIC/ESI+ and 9813 metabolic features from C18/ESI assessments were included in this analysis after data filtering for untargeted analyses.

Targeted analyses were conducted on microbiome-related metabolites with previously confirmed identities [18], including tryptophan metabolites and phenylacetic acid, as well as linoleic acid and related oxylipins, which were previously linked to BMD [15]. All metabolites included in the targeted analyses had a level 1 metabolite identification designation based on criteria outlined by Schymanski and colleagues [22].

### 2.5. Biostatistics and Bioinformatics

Descriptive statistics were conducted for all variables, and the data are presented as the mean ± standard deviation (SD) for continuous variables or count and proportion for categorical variables. Histogram plots for P1NP and CTX were visually inspected for normality and deemed to have a normal distribution. Pearson correlations were used to explore relationships between the BTMs and measures of BMD using JMP Pro (Version 13, SAS Institute Inc, Cary, NC, USA). Metabolomics analyses were performed in RStudio (Boston, MA, USA, Version 1.1.447). Linear regression analyses were used to test for metabolome-wide associations with P1NP and CTX at a *p* < 0.05. Pathway analyses were performed on metabolic features significantly associated with P1NP and CTX using mummichog software (version 2.0.6, www.mummichog.org).

## 3. Results

### 3.1. Characteristics of the Study Population

**Figure 1:**
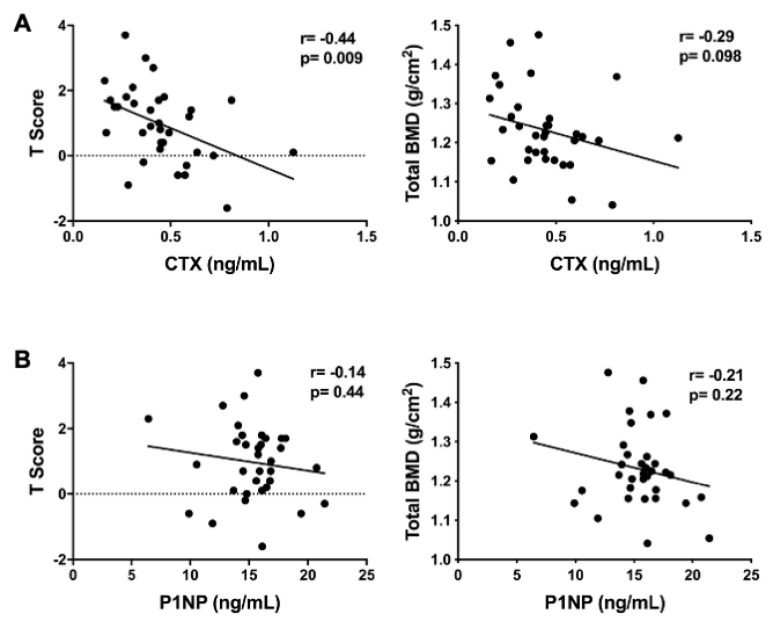
Pairwise associations of the serum bone turnover markers with measures of bone density. (**A**) Association between C-terminal telopeptides of type I collagen (CTX) with whole body BMD T-score and total bone mineral density (BMD); (**B**) association between procollagen type I N-terminal propeptide (P1NP) with whole body BMD T-score and total BMD.

Participants were 34 healthy men (44%) and women (56%) with an average age of 27.8 years (Table 1). The average participant BMI was within the normal weight category (24.7 kg/m^2^) and ranged from 18.6 to 36.5 kg/m^2^. The participants had a normal total BMD and T-score (Table 1). About half of participants reported taking a multivitamin. One participant reported the use of a calcium supplement, and two participants reported taking a vitamin D supplement. The average concentrations for P1NP and CTX were 15.4 ± 2.9 ng/mL and 0.45 ± 0.20 ng/mL, respectively. There was a significant, negative association between CTX and BMD T-scores (*r* = −0.44; *p* = 0.009) and there was a trend towards significance between CTX concentrations and total BMD (*r* = −0.29; *p* = 0.098) (Figure 1A). P1NP was not significantly associated with BMD T-scores or total BMD (Figure 1B).

### 3.2. Metabolomic Associations with P1NP

**Figure 2:**
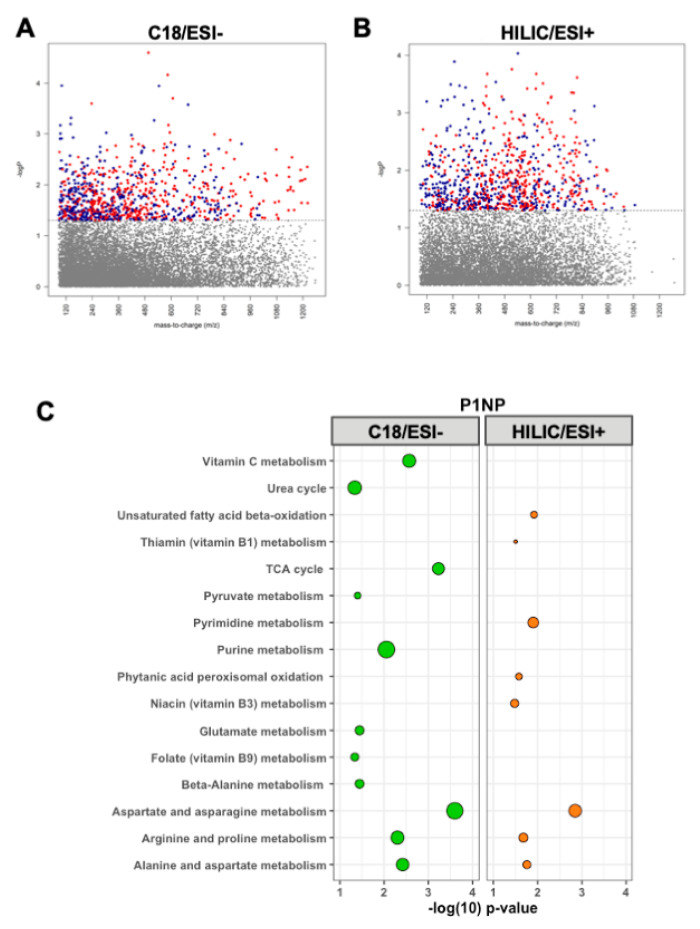
Manhattan plots depicting metabolic features by their mass-to-charge ratio (*m*/*z*) significantly associated with (red = inverse relationship, blue = positive relationship) procollagen type I N-terminal propeptide (P1NP) using (**A**) C18/ESI and (**B**) HILIC/ESI+ high-resolution metabolomics data. Hydrophilic interaction liquid chromatography (HILIC) in positive electrospray ionization (ESI, HILIC/ESI+) mode and reverse phase (C18) chromatography in negative ESI mode (C18/ESI-). (**C**) The pathway analyses of the 847 metabolic features from C18 and 881 metabolic features from HILIC+ significantly associated (*p* < 0.05) with P1NP. Larger bubbles indicate a greater number of significantly enriched metabolites within the respective pathway. Green represents C18/ESI- data; orange represents HILIC/ESI+ data.

Serum P1NP levels were significantly correlated with 847 metabolic features detected by C_18_/ESI-chromatography at *p* < 0.05 (Figure 2A). Pathway enrichment analysis performed on these significant metabolic features revealed 11 significantly enriched metabolic pathways (Figure 2C), including several amino acid pathways, the TCA cycle, folate, and vitamin C metabolism.

A total of 881 metabolic features detected by HILIC/ESI+ chromatography were associated with P1NP at *p* < 0.05 (Figure 2B). Pathway enrichment analyses detected eight significantly enriched pathways, including fatty acid beta-oxidation, thiamin, niacin, pyrimidine, and metabolism of amino acids, which overlapped with the C_18_/ESI pathway enrichment results (Figure 2C).

### 3.3. Metabolomic Associations with CTX

**Figure 3:**
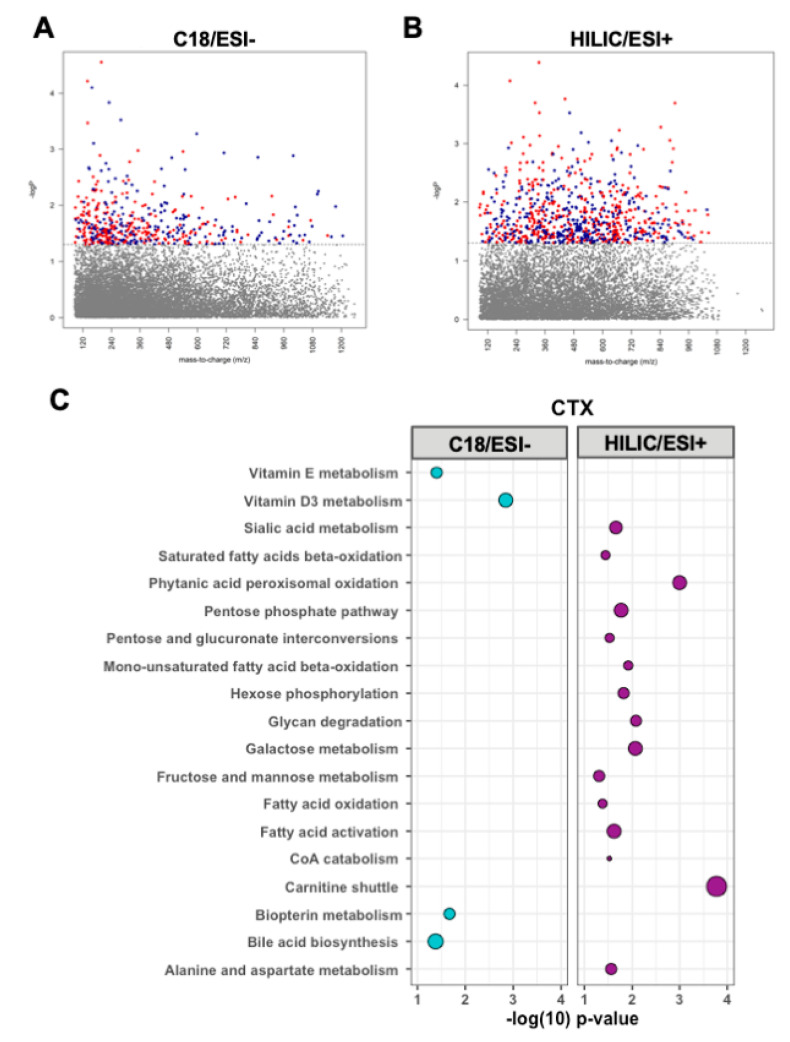
Manhattan plots depicting metabolic features by their mass-to-charge ratio (*m*/*z*) significantly associated with (red = inverse relationship, blue = positive relationship) C-terminal telopeptides of type I collagen (CTX) using (**A**) C18/ESI and (**B**) HILIC/ESI+ high-resolution metabolomics data. (**C**) Pathway analyses of the 790 metabolic features from C18 and 469 metabolic features from HILIC+ significantly associated (*p* < 0.05) with CTX. Larger bubbles indicate a greater number of significantly enriched metabolites within the respective pathway. Blue represents C18/ESI- data, purple represents HILIC/ESI+ data.

CTX was associated with 790 metabolic features detected by C_18_/ESI-chromatography at *p* < 0.05 (Figure 3A). Pathway enrichment analysis of these significant metabolic features identified four significantly enriched pathways: vitamin D metabolism as the top significantly enriched pathway, followed by biopterin metabolism, vitamin E metabolism, and bile acid biosynthesis (Figure 3C).

There were 469 metabolic features detected by HILIC/ESI+ chromatography associated with CTX at *p* < 0.05 (Figure 3B). Fifteen metabolic pathways were significantly enriched within the HILIC/ESI+ metabolites associated with CTX. These pathways were primarily pathways of fatty acid metabolism, including carnitine shuttle, fatty acid activation, CoA metabolism, and fatty acid beta-oxidation, and pathways of carbohydrate metabolism, including fructose and mannose metabolism, hexose phosphorylation, and galactose metabolism as well as alanine and aspartate metabolism (Figure 3C).

### 3.4. Targeted Analyses of Plasma Metabolites

Results of targeted analyses specific to microbiome and linoleic acid-related metabolites previously related to BMD are shown in Table 2. Serum P1NP concentrations were positively associated with the tryptophan metabolites 3-methyl-2-oxindole levels (0.16 ± 0.08, *p* = 0.04) and indole-3-acetylaldehyde (0.04 ± 0.02, *p* = 0.01). An inverse relationship of P1NP with tryptophan (−0.03 ± 0.02, *p* = 0.07) and kynurenine levels (−0.03 ± 0.02, *p* = 0.06) approached significance. CTX concentrations were positively associated with indole-3-ethanol (1.47 ± 0.68, *p* = 0.04) and inversely related to phenylacetate levels (−1.02 ± 0.48, *p* = 0.04). CTX and linoleic acid levels were positively associated, though not statistically significant (1.88 ± 1.04, *p* = 0.08).

## 4. Discussion

Peak bone mass, achieved around 30 years of age, is one of the most powerful predictors of bone mass and fracture risk later in life [23]. Identifying the metabolic pathways associated with sensitive biomarkers of bone formation and resorption during this critical period of bone development provides valuable insight into nutrition-related pathways that could be targeted to improve bone health and prevent osteoporosis. In this population of healthy young adults, we identified several distinct plasma metabolic pathways associated with the reliable BTMs, P1NP and CTX. P1NP was predominantly related to amino acid and B vitamin metabolism, whereas CTX was associated with lipid-related pathways and fat-soluble vitamin metabolism, including vitamin D and vitamin E.

In this study, the bone formation biomarker P1NP was associated with several metabolic pathways related to amino acid, vitamin C, B vitamins, and central energy (e.g., TCA cycle, pyruvate metabolism) metabolism. Vitamin C was one of the top pathways associated with P1NP. Vitamin C plays a critical role in the development and maintenance of bone where it increases the rates of both procollagen hydroxylation and secretion. Prior work has confirmed the importance of vitamin C in maintaining bone health by demonstrating a positive association with vitamin C intake and BMD at the femoral neck and lumbar spine along with a 33% lower risk of osteoporosis [24]. These effects may stem from the suppression of osteoclast activity and the differentiation of osteoblasts by vitamin C [25,26]. In this regard, the association between vitamin C metabolism and P1NP levels may reflect the well-established osteogenic effects of vitamin C on osteoblast differentiation and matrix deposition [27].

Previous metabolomics-based studies have reported associations with amino acids and assessments of bone health [28,29,30]. Additional work has linked the intake of alanine, arginine, glutamic acid, and proline to a higher BMD at the spine and forearm in a cross-sectional study of females aged 18 to 79 years [31]. In line with these previous reports, P1NP concentrations were positively related to several amino acids and their metabolic pathways, including alanine, aspartate, arginine, proline, glutamate, and beta-alanine. P1NP is the by-product of type I procollagen processing into type I collagen, which is comprised of approximately 23% of proline/hydroxyproline [32]. Proline can also be biosynthetically derived from arginine and glutamate [32]. These relationships may arise from the requirement of specific amino acids to facilitate collagen formation. Beyond the potential effects on collagen synthesis, arginine enhances osteoblastogenesis and increases type I collagen production [33,34,35]. These significant associations between P1NP and specific amino acids suggests that amino acid metabolism may be important for osteoblast formation and function, but future studies are ultimately needed to test the relationships between specific amino acids and levels of the surrogate markers of osteoblast activity P1NP.

Our study identified distinct energy-yielding metabolic pathways associated with the surrogate markers of bone formation (P1NP) and bone resorption (CTX). P1NP was associated with central metabolic pathways such as the TCA cycle, pyruvate metabolism, and metabolism of B vitamins important for energy production (i.e., niacin, thiamin). Conversely, CTX was associated with lipid and fatty acid beta-oxidation metabolic pathways. The modeling and remodeling of bone consumes significant quantities of ATP to facilitate the synthesis of new collagen by osteoblasts [36]. Osteoblasts preferentially utilize glucose to meet these high energetic needs [37,38,39,40]. Aerobic glycolysis, in particular, is important for providing metabolic intermediates to support the production of collagen [41].

Bone resorption is also metabolically expensive; we found that serum CTX concentrations were associated with lipid and fatty acid metabolic pathways rather than carbohydrate metabolism. Fatty acids are transported into the mitochondria via the carnitine shuttle where they undergo beta-oxidation to generate acetyl-CoA that then feeds into the TCA cycle to generate ATP. Actively resorbing osteoclasts demonstrate a high capacity for fatty acid beta-oxidation that is likely facilitated through the high number of mitochondria within osteoclasts [42,43,44,45,46,47]. The associations we found between fatty acid metabolism and CTX are intriguing as they suggest that the high-energetic state of active osteoclasts may be principally supported by high energy-yielding lipid catabolism. To date, most studies focusing on osteoclast metabolism have limited their analyses to carbohydrate metabolism [48]. Our study suggests that lipid metabolism may also be important for osteoclast activity and should be further investigated in future studies.

Fat-soluble vitamin metabolism emerged as a predominant correlate with serum CTX levels, with vitamin D metabolism emerging as the top pathway associated with CTX in our study. Corroborating our findings, a previous study reported that vitamin D (25(OH)D) is positively correlated with serum CTX concentrations [49]. Mechanistically, this association may stem from the vitamin D-induced secretion of RANKL from osteoblasts, which would lead to increased osteoclastic bone resorption and a resultant increase in serum CTX levels [50,51,52]. Vitamin E metabolism was also associated with CTX. Vitamin E is a fat-soluble vitamin that has been previously been shown to be negatively correlated with the femoral neck bone mineral density [53]. These effects may be due to the effect of vitamin E on osteoclast fusion thereby leading to decreased bone mass [54]. Collectively, these findings indicate that fat-soluble vitamins may contribute to bone metabolism as reflected by BTMs.

Metabolites within the bile acid biosynthesis pathway were positively associated with serum CTX levels. This intriguing relationship between bile acid metabolism and bone metabolism has also been previously identified in a young population (20–40 years of age) of US Caucasian women, where metabolomics analyses were used to identify a significant association between several bile acids (cholic acid, ursodeoxycholic acid, tauroursodeoxycholic acid) and increased risk for low hip BMD [29]. The role of bile acids in bone turnover is complex and not well-defined. For instance, the bile acid lithocholic acid can upregulate the expression of RANKL in osteoblasts that may enhance the formation of osteoclasts within the basic multicellular unit [55], whereas global deletion of the bile acid receptor (FXR) in mice increased the number and size of osteoclasts [56]. Considering that bile acids are critically important for the emulsification and absorption of fatty acids and fat-soluble vitamins (e.g., vitamin D, vitamin E), the association between bile acid metabolism and CTX may be partly driven by the other lipid-related metabolic pathways associated with CTX. Additional studies are ultimately needed to fully define the influence of bile acid metabolism on bone resorption.

Targeted analyses identified associations with BTMs and microbiota-related metabolites. In line with our previous findings of metabolomic associations with BMD, two tryptophan-derived metabolites were significantly, positively related to bone formation as measured by P1NP [15]. Conversely, another tryptophan metabolite, indole-3-enthanol, was also positively associated with CTX levels. Tryptophan metabolism has been proposed to influence osteoclast activity [13] and, through kynurenines and bacterial tryptophan metabolites acting as aryl hydrocarbon (AhR) ligands, may regulate immune response and T cell function [57,58]. These findings add to the growing body of literature linking the intestinal microbiota to bone development and metabolism [59,60,61,62,63,64,65,66,67]. Future work should link BTMs with data characterizing the gut microbiome of participants in a longitudinal manner.

The present study has several notable strengths. HRM provides broad chemical detection to study metabolites associated with BTMs in an unbiased, hypothesis-free approach. Additionally, this study combined data from HILIC and C18 chromatography that maximized the detection of serum metabolites, and we utilized both targeted and unbiased, untargeted analyses. The use of BTMs also provides a sensitive assessment of bone metabolism. There are, however, several limitations of the current study, including a moderate sample size consisting of mostly Caucasian subjects recruited from a single center. Due to the modest sample size of our study, we were not able to explore the influence of sex and race on BTMs and plasma metabolic pathways. Both sex and race/ethnicity are well-established factors that influence bone metabolism [27,68,69] and may also influence the relationship between BTMs and specific nutrients. Replicating this study in a larger, prospective, and heterogenous population would allow for stratified analyses based on demographic characteristics and would further corroborate our findings. The cross-sectional design of this study is also a limitation as it does not allow for casual inferences to be made regarding the detected metabolites and BTMs. Lastly, the DXA assessment in this study was a total body scan and including site-specific measures of bone density, such as the lumbar spine and femoral neck, would benefit future work as these locations are prone to fractures.

## 5. Conclusions

This study provides important hypothesis-generating data for investigating metabolites and metabolic pathways that reflect bone formation and bone resorption. Nutrition-related pathways, including fatty acids, amino acids, and carbohydrate metabolism were diversely related to P1NP and CTX concentrations. Characterizing these pathways associated with BTMs in healthy adults who are acquiring peak bone mass is an important step towards understanding the metabolic perturbations that lead to low bone mass in clinical and older adult populations. Future longitudinal studies should focus on replicating these findings in different populations, such as post-menopausal women, elderly populations, or individuals with chronic diseases. Moreover, clinical studies are ultimately warranted to determine if targeting the identified metabolic pathways using dietary interventions can positively modify BTMs and improve overall bone health.

## Figures and Tables

**Table 1:** Demographic characteristics (*N* = 34).

Characteristic	*n* (%) or Mean ± SD	Range
Female *n* (%)	19 (56)	-
Race *n* (%)		
Caucasian	20 (59)	-
Asian	11 (32)	-
African American	3 (9)	-
Age (years)	27.8 ± 4.9	18–36
BMI	24.7 ± 4.0	18.6–36.5
Body Fat (%)		
Females	31.1 ± 7.3	17.0–48.1
Males	24.1 ± 8.6	9.8–40.3
Total BMD (gm/cm^2^)	1.23 ± 0.10	1.04–1.48
Whole-Body BMD T-score	0.97 ± 1.2	−1.6–3.7
Multivitamin supplement	15 (44)	-
Calcium supplement	1 (3)	-
Vitamin D supplement	2 (6)	-
P1NP (ng/mL)	15.4 ± 2.9	6.43–21.42
CTX (ng/mL)	0.45 ± 0.20	0.16–1.13

Abbreviations: SD, standard deviation; BMI, body mass index; BMD, bone mineral density P1NP, Procollagen type I N-terminal propeptide; CTX, C-terminal telopeptides of Type I collagen. -: Not applicable to put a range for non-continuous variables, could be left blank.

**Table 2:** Linear regression analyses of microbiome-related and linoleic acid-related plasma metabolites (dependent variable) related to bone turnover markers (independent variable).

Plasma Metabolite	*m*/*z*	Time (s)	β Estimate ± SE	*p*-Value
**Associations with P1NP**				
Tryptophan	205.0971	36	−0.03 ± 0.02	0.07
3-Hydroxykynurenine	225.0823	33	−0.02 ± 0.05	0.76
L-Kynurenine	209.092	40	−0.05 ± 0.03	0.06
Indole	118.0652	38	−0.03 ± 0.02	0.11
Indole-3-Acetaldehyde	160.0798	90	0.04 ± 0.02	**0.01**
5-Hydroxyindoleacetate	192.0655	38	–0.08 ± 0.05	0.09
Indole-3-Ethanol	162.0955	57	0.08 ± 0.05	0.14
3-Methyl-2-Oxindole	148.0732	248	0.16 ± 0.08	**0.04**
Indole-3-Acetic Acid	176.0705	24	–0.1 ± 0.07	0.17
Methyl Indole-3-Acetate	190.0845	153	–0.14 ± 0.11	0.20
Phenylacetic Acid	137.0551	68	0.03 ± 0.04	0.40
Linoleic Acid (FA 18:2)	281.2475	199	0.01 ± 0.08	0.90
HPODE	313.2373	259	–0.01 ± 0.05	0.80
HODE/EpOME	297.2211	221	–0.03 ± 0.04	0.50
**Associations with CTX**				
Tryptophan	205.0971	36	0.02 ± 0.24	0.93
3-Hydroxykynurenine	225.0823	33	–0.56 ± 0.7	0.43
L-Kynurenine	209.092	40	0.37 ± 0.38	0.33
Indole	118.0652	38	0.22 ± 0.24	0.36
Indole-3-Acetaldehyde	160.0798	90	0.36 ± 0.25	0.16
5-Hydroxyindoleacetate	192.0655	38	0.47 ± 0.66	0.48
Indole-3-Ethanol	162.0955	57	1.47 ± 0.68	**0.04**
3-Methyl-2-Oxindole	148.0732	248	1.48 ± 0.45	0.58
Indole-3-Acetic Acid	176.0705	24	0.0001 ± 0.99	1.00
Methyl Indole-3-Acetate	190.0845	153	0.65 ± 1.16	0.33
Phenylacetic Acid	137.0551	68	1.02 ± 0.48	**0.04**
Linoleic Acid (FA 18:2)	281.2475	199	1.88 ± 1.04	0.08
HPODE	313.2373	259	0.64 ± 0.64	0.33
HODE/EpOME	297.2211	221	0.64 ± 0.6	0.29

Bolded *p*-values indicate significant findings at *p* < 0.05. All metabolites were matched to positive ions and have a level 1 metabolite identification confidence score. Abbreviations: *m*/*z*, mass-to-charge ratio; SE, standard error; HPODE, hydroperoxy-octadecadienoic acid; EpOME, epoxyoctadecenoic acid (a peroxidation product of linoleic acid); HODE, hydroxyoctadecadienoic acid (a derivative of linoleic acid).
